# Supplementary material for: A new global ice sheet reconstruction for the past 80 000 years
Source: Nat Commun. 2021 Feb 23;12:1199. doi: 10.1038/s41467-021-21469-w (PMC7902671; doi:10.1038/s41467-021-21469-w)
Supplement: Supplementary file 1 — Supplementary Information [file 41467_2021_21469_MOESM1_ESM.pdf]

A new global ice sheet reconstruction for the past 80 000 years - Supplementary Information

Evan J. Gowan[1,2], Xu Zhang[1,3], Sara Khosravi[4], Alessio Rovere[2], Paolo Stocchi[5], Anna L. C. Hughes[6,7], Richard Gyllencreutz[8], Jan Mangerud[7], John-Inge Svendsen[7] & Gerrit Lohmann[1,2]

[1]*Alfred Wegener Institute, Helmholtz Center for Polar and Marine Research, Bremerhaven, Germany*

[2]*MARUM, University of Bremen, Bremen, Germany*

[3]*Key Laboratory of Western China's Environmental Systems (Ministry of Education), College of Earth and Environmental Science, Centre for Pan Third Pole Environment (Pan-TPE), Langzhou University, Langzhou, China*

[4]*Alfred Wegener Institute, Helmholtz Center for Polar and Marine Research, Potsdam, Germany*

[5]*NIOZ, Texel, Netherlands*

[6]*Department of Geography, University of Manchester, Manchester, United Kingdom*

[7]*Department of Earth Science, University of Bergen and Bjerknes Centre for Climate Research, Bergen, Norway*

[8]*Department of Geological Sciences, Stockholm University, Stockholm, Sweden*

This supplement contains figures showing the global paleo-topography at 42 500 and 60 000 yr BP, as well as calculated sea level at locations with MIS 3 aged sea level indicators. Paleo-topography plots for all of the time slices are incluced in the dataset on Pangaea (`https://doi.org/10.1594/PANGAEA.905800`). The sea level indicators include index points (sea

level is near or at the elevation of the sample), terrestrial limiting (sea level should be below the elevation of the sample), and marine limiting (sea level should be above the elevation of the sample). There are also figures showing how the paleo-sea level in Churchill (south of Hudson Bay) and Ångermanland (on the Swedish coast of the Baltic Sea) to show the results of using different lower mantle viscosity values, and higher temporal resolution than the standard version of PaleoMIST 1.0.

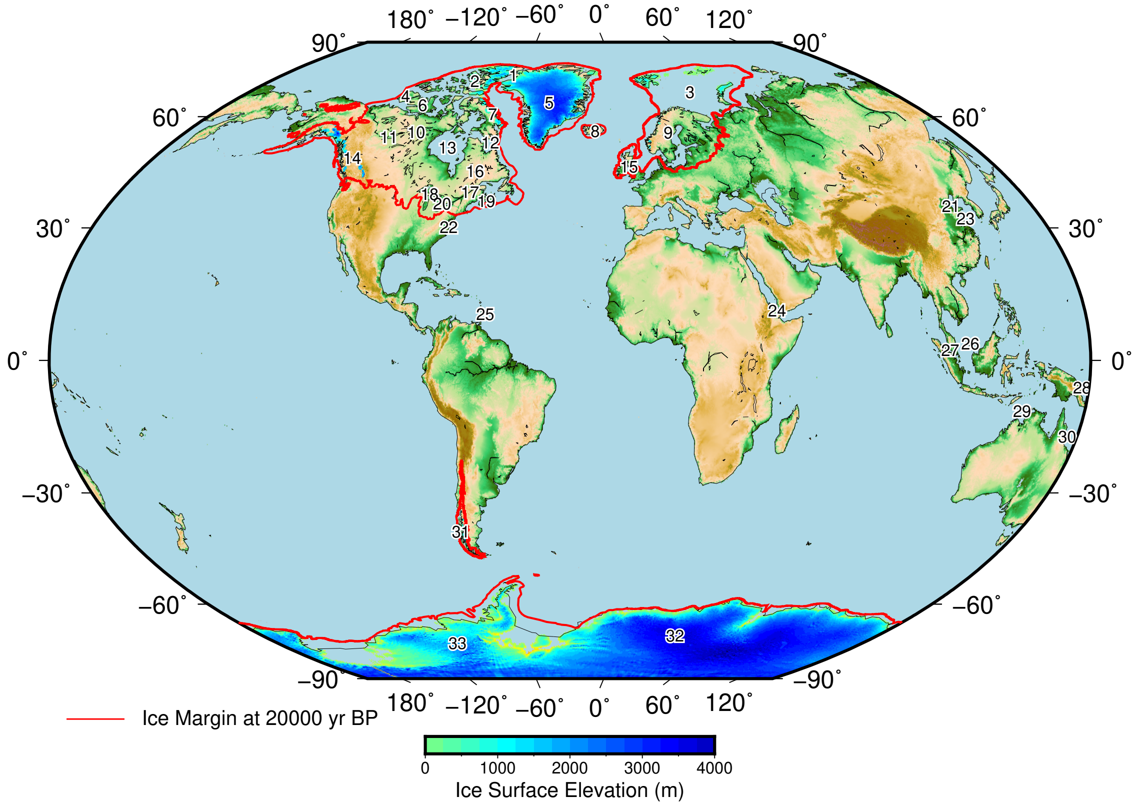

Supplementary Figure 1: Location map with modern topography. The red lines show the ice sheet margins at 20 000 yr BP, the Last Glacial Maximum. 1 - Nares Strait; 2 - Innuitian Ice Sheet; 3 - Eurasia: Svalbard-Barents-Kara Ice Sheet; 4 - Banks Island; 5 - Greenland Ice Sheet; 6 - Victoria Island; 7 - Baffin Island; 8 - Iceland; 9 - Eurasia: Scandinavian Ice Sheet; 10 - Laurentide Ice Sheet - Keewatin dome; 11 - Great Slave Lake; 12 - Hudson Strait; 13 - Hudson Bay; 14 - Cordilleran Ice Sheet; 15 - Eurasia: British-Irish Ice Sheet; 16 - Laurentide Ice Sheet - Quebec/Labrador dome; 17 - St. Lawrence Valley; 18 - Lake Superior/Michigan; 19 - Nova Scotia; 20 - Southern Ontario; 21 - Bohai Sea; 22 - Eastern United States; 23 - Yellow Sea; 24 - Red Sea proxy record; 25 - Barbados; 26 - Sunda Shelf; 27 - Strait of Malacca; 28 - Huon Peninsula; 29 - Bonaparte Gulf; 30 - Great Barrier Reef - Cairns; 31 - Patagonia Ice Sheet; 32 - East Antarctic Ice Sheet; 33 - West Antarctic Ice Sheet

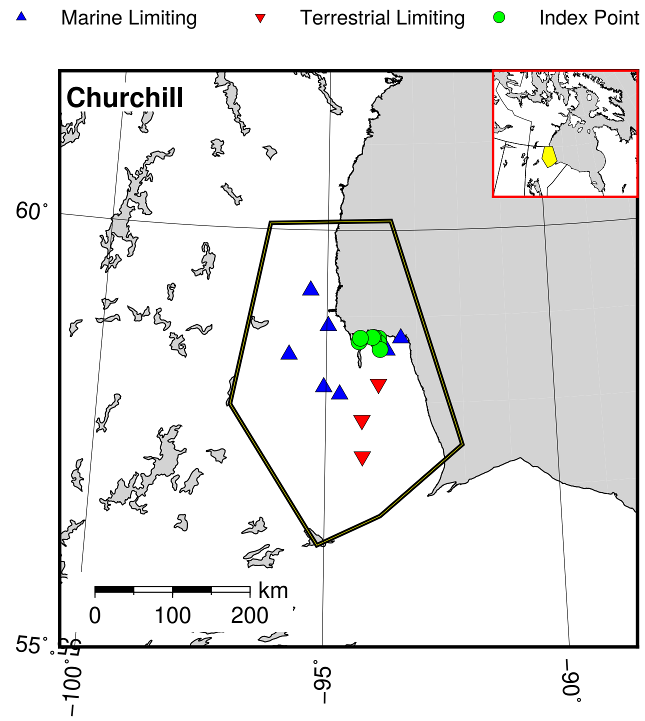

Supplementary Figure 2: Map showing the location of sea level indicators in the Churchill region of southern Hudson Bay[1].

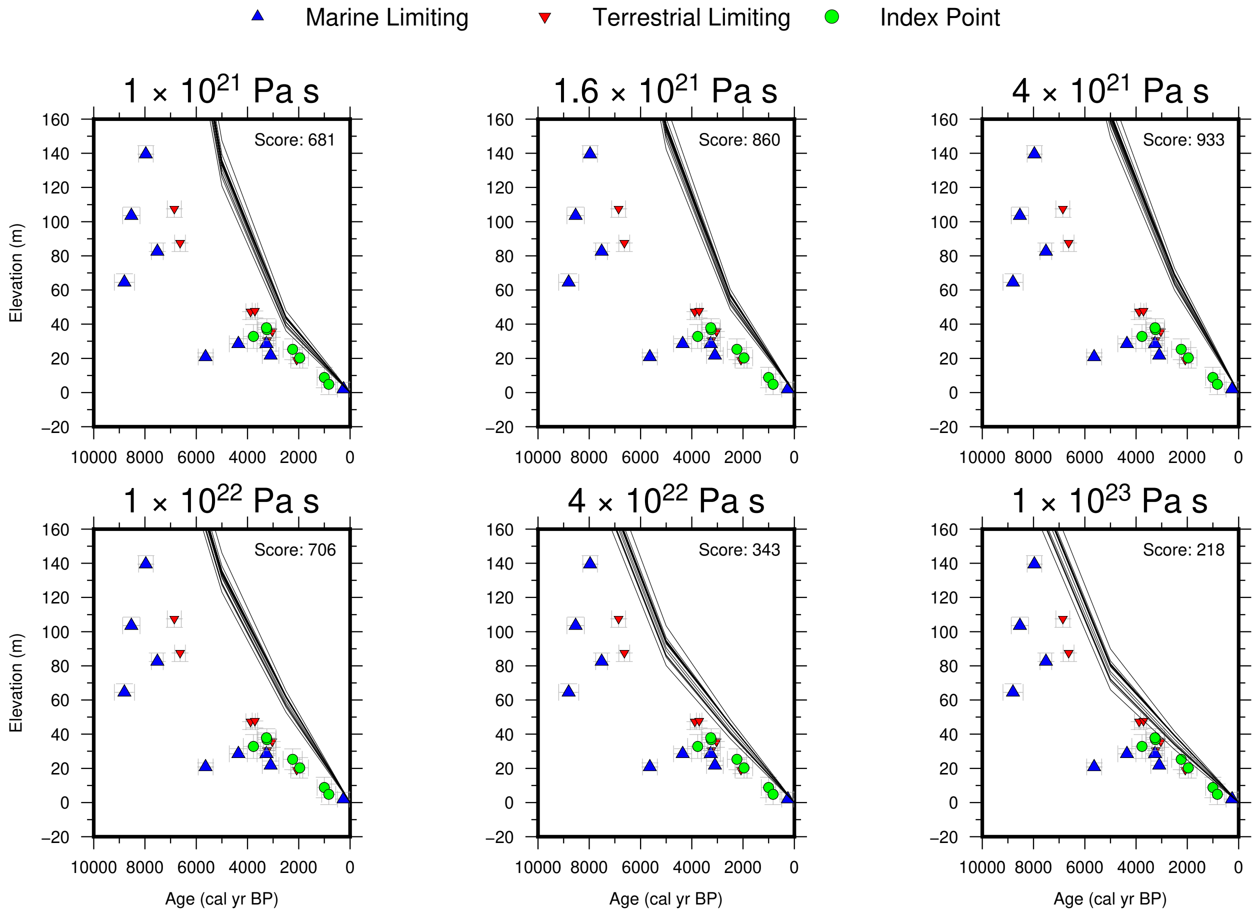

Supplementary Figure 3: Sea level indicators in the Churchill region of southern Hudson Bay[1] and calculated sea level at the location of each indicator (the spread in the response is due to spatial variations in calculated sea level). The calculated sea level is lower using a higher lower mantle viscosity and therefore closer to the sea level infered from the sea level indicators. Using a higher lower mantle viscosity therefore makes it easier to simulaneously include larger ice volumes in central Canada and fit the sea level indicators. Note that the calculated sea level is still too high, which is at least partly a consequence of the low temporal resolution of the reconstruction and the fact that SELEN treats the load between time steps as a heaviside function. This will cause the load to be overestimated during deglaciation, a factor that will be mitigated in future studies when a higher temporal resolution will be used.

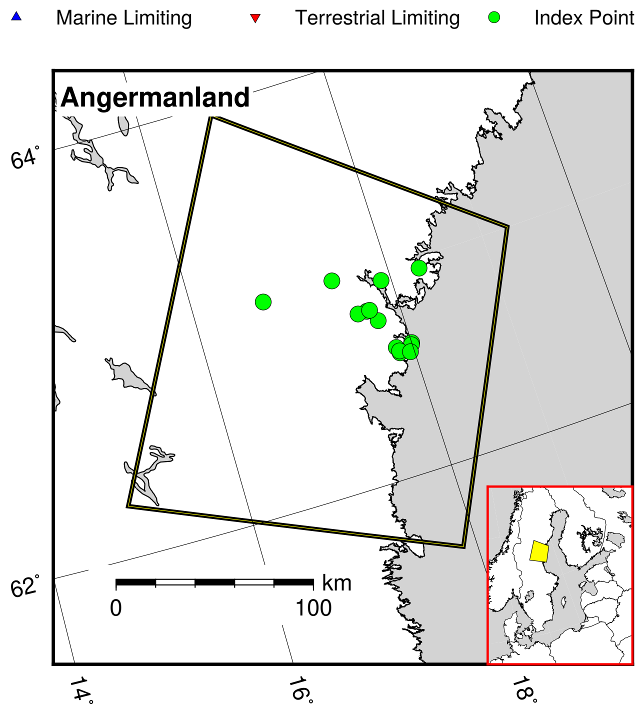

Supplementary Figure 4: Map showing the location of sea level indicators in the Ångermanland in the Baltic Sea (data from an upcoming compilation by Rosentau *et al.*)

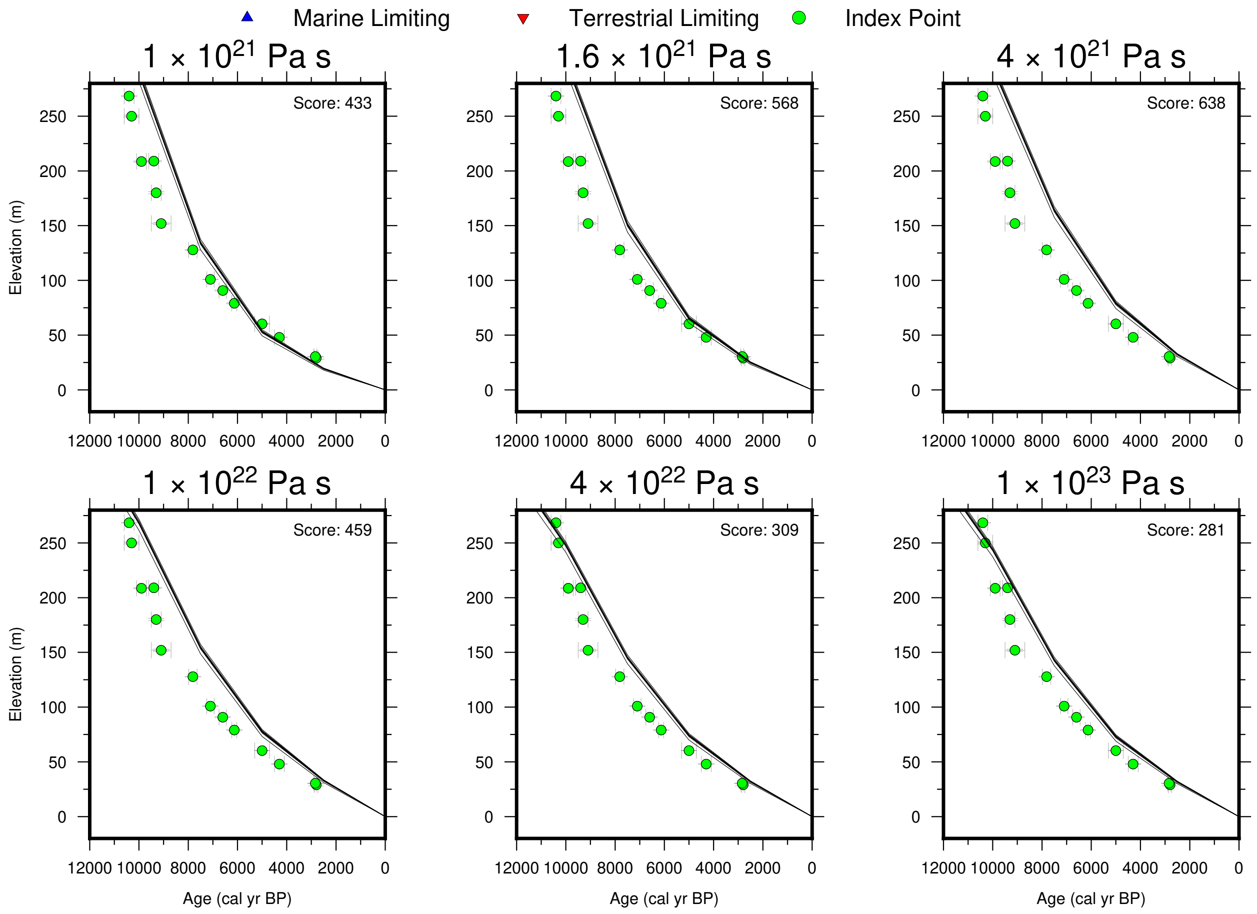

Supplementary Figure 5: Sea level indicators in the Ångermanland in the Baltic Sea (data from an upcoming compilation by Rosentau *et al.*) and calculated sea level at the location of each indicator (the spread in the response is due to spatial variations in calculated sea level). The calculated sea level is lower using a higher lower mantle viscosity and therefore closer to the sea level infered from the sea level indicators. Although it is not expected that the lower mantle is sensitive to the Eurasian Ice Sheet, the response is improved by using the higher lower mantle viscosity. The response may be affected by the North American ice sheets[2]. Note that the calculated sea level is still too high, which is at least partly a consequence of the low temporal resolution of the reconstruction and the fact that SELEN treats the load between time steps as a heaviside function. This will cause the load to be overestimated during deglaciation, a factor that will be mitigated in future studies when a higher temporal resolution will be used.

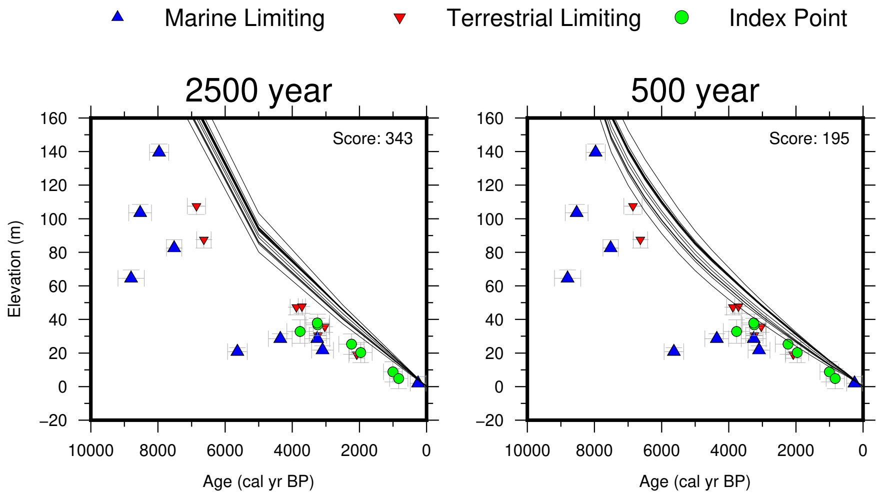

Supplementary Figure 6: Sea level indicators in the Churchill region of southern Hudson Bay[1] and calculated sea level at the location of each indicator (the spread in the response is due to spatial variations in calculated sea level). This plot shows the difference between the standard version of PaleoMIST 1.0 with 2500 year time steps, and a model run where the load is linearly interpolated to 500 year time steps. The higher temporal resolution better captures the deglaciation, and the calculated sea level is closer to the observations.

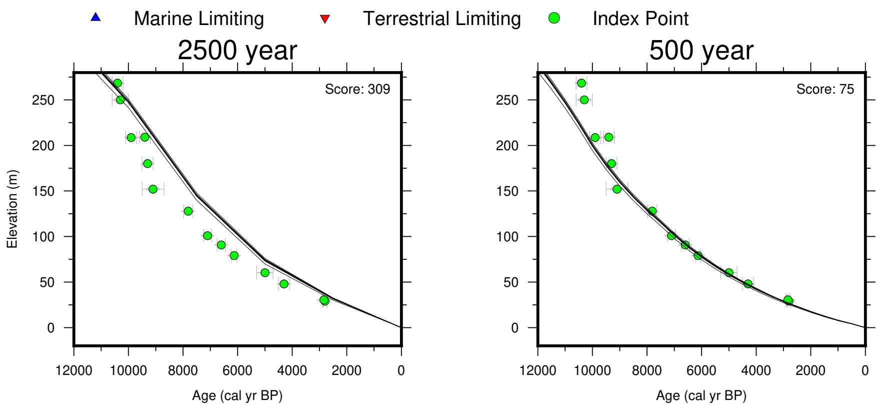

Supplementary Figure 7: Sea level indicators in the Ångermanland in the Baltic Sea (data from an upcoming compilation by Rosentau *et al.*) and calculated sea level at the location of each indicator (the spread in the response is due to spatial variations in calculated sea level). This plot shows the difference between the standard version of PaleoMIST 1.0 with 2500 year time steps, and a model run where the load is linearly interpolated to 500 year time steps. The higher temporal resolution better captures the deglaciation, and the calculated sea level is closer to the observations.

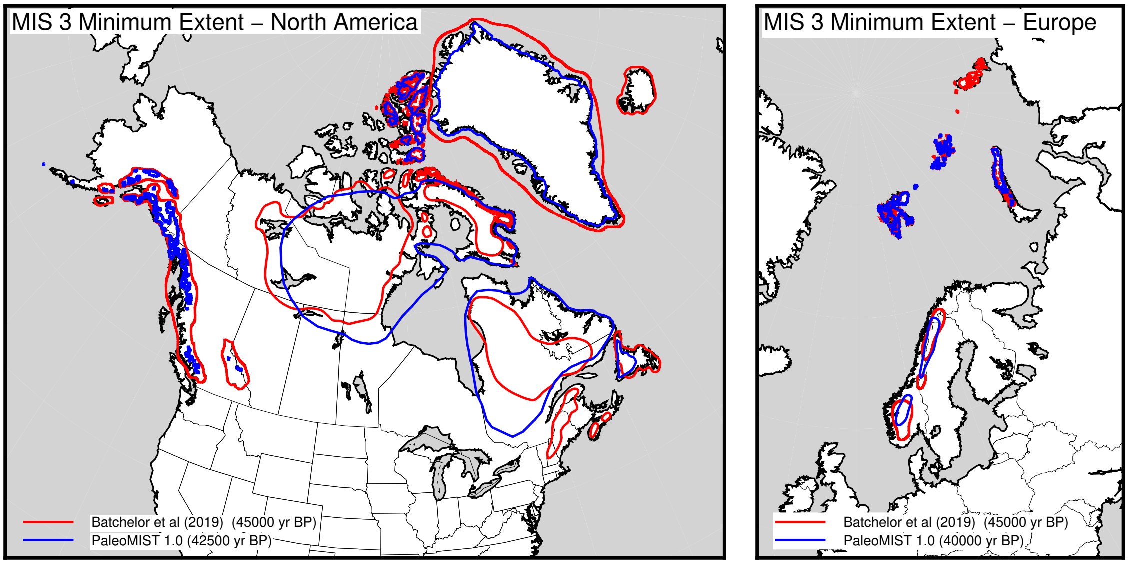

Supplementary Figure 8: Comparison of the MIS 3 minimum extent margin reconstruction by Batchelor *et al.*[3], and PaleoMIST 1.0 for the North America and Eurasian ice sheets. The minimum extent in the Batchelor *et al.* reconstruction is set to 45000 yr BP in their "best estimate" margin set. The minimal ice scenario (with an ice free Hudson Bay) is set to be 42500 yr BP in PaleoMIST 1.0, while the minimum extent of Eurasia is set to 40000 yr BP.

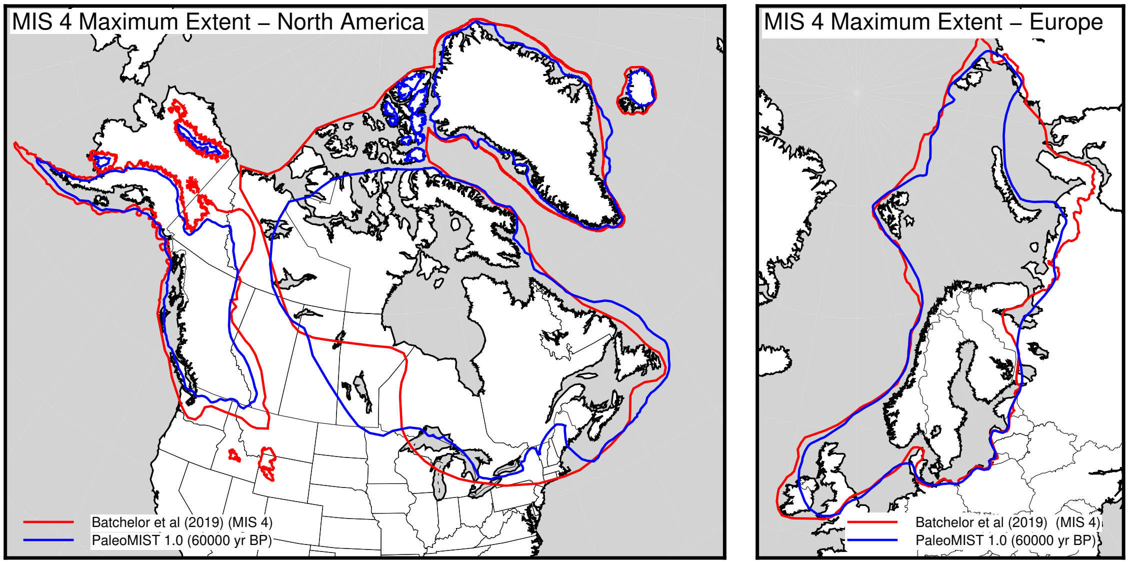

Supplementary Figure 9: Comparison of the MIS 4 maximum extent margin margin reconstruction by Batchelor *et al.*[3], and PaleoMIST 1.0 for the North America and Eurasian ice sheets. The margin displayed here for *et al.* is their "best estimate" margin set. The PaleoMIST 1.0 margin corresponds to 60000 yr BP

Supplementary Table 1: Ice volume and sea level equivalent for the four major ice sheets, from the minimal scenario

Age	North America		Eurasia		Antarctica		Patagonia		Total		Calcualted
(yr BP)	volume (10^6 m^3)	SLE (m)	volume (10^6 m^3)	SLE (m)	volume (10^6 m^3)	SLE (m)	volume (10^6 m^3)	SLE (m)	volume (10^6 m^3)	SLE (m)	ESL (m)
0	0.0	0.0	0.0	0.0	0.0	0.0	0.0	0.0	0.0	0.0	0.0
2500	−0.1	−0.1	0.0	0.0	0.6	1.5	0.0	0.0	0.5	1.4	0.0
5000	−0.1	−0.4	0.0	0.0	1.4	3.5	0.0	0.0	1.2	3.1	−4.6
7500	0.8	2.0	0.0	0.0	1.7	4.4	0.0	0.0	2.5	6.4	−8.4
10000	8.1	20.4	0.0	0.1	2.0	5.1	0.0	0.0	10.2	25.6	−28.2
12500	15.7	39.6	1.1	2.7	3.2	8.0	0.0	0.0	20.0	50.3	−54.1
15000	23.4	59.1	2.7	6.7	3.5	8.8	0.0	0.1	29.6	74.7	−80.3
17500	29.8	75.2	5.4	13.7	3.8	9.5	0.2	0.6	39.2	98.9	−107.1
20000	30.7	77.3	7.3	18.5	3.9	9.7	0.3	0.7	42.2	106.3	−116.0
22500	29.5	74.4	6.5	16.4	4.0	10.2	0.3	0.7	40.4	101.7	−111.4
25000	26.4	66.5	4.3	10.9	4.2	10.5	0.3	0.8	35.2	88.7	−97.3
27500	21.1	53.2	3.2	8.1	4.2	10.5	0.3	0.8	28.8	72.6	−79.7
30000	16.0	40.4	1.7	4.2	4.0	10.1	0.3	0.8	22.0	55.5	−60.9
32500	13.6	34.3	0.9	2.3	3.8	9.5	0.2	0.5	18.5	46.6	−51.2
35000	12.6	31.7	0.4	1.0	2.7	6.7	0.2	0.5	15.8	39.9	−43.4
37500	11.5	29.1	0.2	0.5	2.1	5.4	0.3	0.8	14.2	35.8	−38.5
40000	8.5	21.5	0.1	0.1	1.8	4.5	0.2	0.5	10.5	26.5	−28.4
42500	7.3	18.3	0.3	0.7	1.7	4.3	0.2	0.5	9.4	23.8	−25.3
45000	11.1	28.1	1.2	3.0	1.6	4.0	0.3	0.8	14.2	35.8	−37.7
47500	13.3	33.5	0.4	1.1	1.5	3.8	0.2	0.5	15.4	38.8	−40.6
50000	12.0	30.4	0.2	0.4	1.4	3.7	0.1	0.2	13.7	34.6	−36.2
52500	14.2	35.8	1.6	4.1	1.4	3.5	0.0	0.1	17.3	43.6	−45.6
55000	16.3	41.0	3.7	9.2	1.3	3.4	0.1	0.2	21.3	53.8	−56.2
57500	17.3	43.6	5.8	14.6	1.5	3.8	0.1	0.4	24.8	62.4	−66.4
60000	20.5	51.7	6.7	17.0	1.8	4.4	0.1	0.3	29.1	73.4	−78.9
62500	17.9	45.0	6.1	15.4	1.7	4.2	0.2	0.5	25.9	65.2	−70.3
65000	11.3	28.4	5.4	13.7	1.7	4.2	0.2	0.5	18.6	46.8	−50.7
67500	7.7	19.4	4.7	11.9	1.5	3.7	0.1	0.2	14.0	35.2	−37.9
70000	5.1	13.0	3.5	8.9	1.3	3.2	0.0	0.0	10.0	25.1	−26.8
72500	3.5	8.7	2.3	5.9	1.3	3.3	0.0	0.0	7.1	17.8	−19.3
75000	2.3	5.8	0.9	2.2	1.3	3.3	0.0	0.0	4.5	11.3	−12.6
77500	1.2	3.1	0.3	0.7	1.4	3.4	0.0	0.0	2.9	7.2	−8.5
80000	1.9	4.8	0.1	0.1	1.3	3.4	0.0	0.0	3.3	8.3	−9.5

ESL is calculated by dividing the water equivalent ice volume by the modern ocean area[4] $V_i \times 0.91/(361 \times 10^6 \mathrm{km}^2)$

Supplementary Table 2: Ice volume and sea level equivalent for the four major ice sheets, from the maximal scenario

Age	North America		Eurasia		Antarctica		Patagonia		Total		Calcualted
(yr BP)	volume (10^6 m^3)	SLE (m)	volume (10^6 m^3)	SLE (m)	volume (10^6 m^3)	SLE (m)	volume (10^6 m^3)	SLE (m)	volume (10^6 m^3)	SLE (m)	ESL (m)
0	0.0	0.0	0.0	0.0	0.0	0.0	0.0	0.0	0.0	0.0	0.0
2500	−0.1	−0.1	0.0	0.0	0.6	1.5	0.0	0.0	0.5	1.3	0.0
5000	−0.1	−0.4	0.0	0.0	1.4	3.5	0.0	0.0	1.2	3.1	−4.6
7500	0.8	2.0	0.0	0.0	1.8	4.5	0.0	0.0	2.6	6.5	−8.4
10000	8.1	20.4	0.0	0.1	2.0	5.1	0.0	0.0	10.2	25.7	−28.2
12500	15.7	39.7	1.1	2.7	3.1	7.9	0.0	0.0	20.0	50.3	−54.2
15000	23.5	59.2	2.7	6.7	3.5	8.8	0.0	0.1	29.7	74.8	−80.4
17500	29.9	75.4	5.4	13.7	3.8	9.5	0.2	0.6	39.3	99.1	−107.3
20000	30.8	77.6	7.3	18.5	3.9	9.9	0.3	0.8	42.3	106.7	−116.4
22500	29.6	74.7	6.5	16.4	4.0	10.2	0.3	0.7	40.4	102.0	−111.6
25000	26.5	66.8	4.3	10.9	4.2	10.6	0.3	0.8	35.3	89.0	−97.6
27500	21.3	53.6	3.2	8.1	4.2	10.6	0.3	0.7	29.0	73.0	−80.1
30000	16.2	40.9	1.7	4.2	4.0	10.1	0.3	0.8	22.2	55.9	−61.3
32500	13.7	34.6	0.9	2.3	3.8	9.5	0.2	0.5	18.6	46.9	−51.5
35000	12.6	31.8	0.4	1.0	2.7	6.7	0.2	0.5	15.9	40.0	−43.6
37500	13.7	34.5	0.2	0.5	2.1	5.4	0.3	0.8	16.3	41.2	−44.0
40000	12.5	31.6	0.1	0.1	1.7	4.3	0.2	0.5	14.5	36.5	−38.5
42500	11.6	29.2	0.3	0.7	1.6	4.1	0.2	0.5	13.7	34.5	−36.3
45000	14.7	37.0	1.2	3.0	1.5	3.9	0.3	0.8	17.7	44.6	−46.6
47500	14.9	37.5	0.4	1.1	1.5	3.8	0.2	0.5	17.0	42.9	−44.6
50000	13.3	33.6	0.2	0.4	1.4	3.6	0.1	0.2	15.0	37.7	−39.4
52500	15.0	37.9	1.6	4.1	1.4	3.5	0.0	0.1	18.1	45.6	−47.7
55000	16.7	42.1	3.7	9.2	1.3	3.4	0.1	0.2	21.8	54.9	−57.3
57500	17.3	43.6	5.8	14.6	1.5	3.8	0.1	0.4	24.7	62.4	−66.3
60000	20.5	51.6	6.7	17.0	1.8	4.4	0.1	0.3	29.1	73.3	−78.9
62500	17.8	45.0	6.1	15.4	1.7	4.2	0.2	0.5	25.8	65.1	−70.2
65000	11.3	28.4	5.4	13.7	1.7	4.2	0.2	0.5	18.5	46.7	−50.7
67500	7.7	19.4	4.7	11.9	1.5	3.7	0.1	0.2	14.0	35.2	−37.9
70000	5.1	13.0	3.5	8.9	1.3	3.2	0.0	0.0	10.0	25.1	−26.7
72500	3.5	8.7	2.3	5.9	1.3	3.2	0.0	0.0	7.1	17.8	−19.2
75000	2.3	5.8	0.9	2.2	1.3	3.3	0.0	0.0	4.5	11.3	−12.6
77500	1.2	3.1	0.3	0.7	1.3	3.4	0.0	0.0	2.8	7.2	−8.4
80000	1.9	4.8	0.1	0.1	1.3	3.3	0.0	0.0	3.3	8.2	−9.4

ESL is calculated by dividing the water equivalent ice volume by the modern ocean area[4] $V_i \times 0.91/(361 \times 10^6 \text{km}^2)$

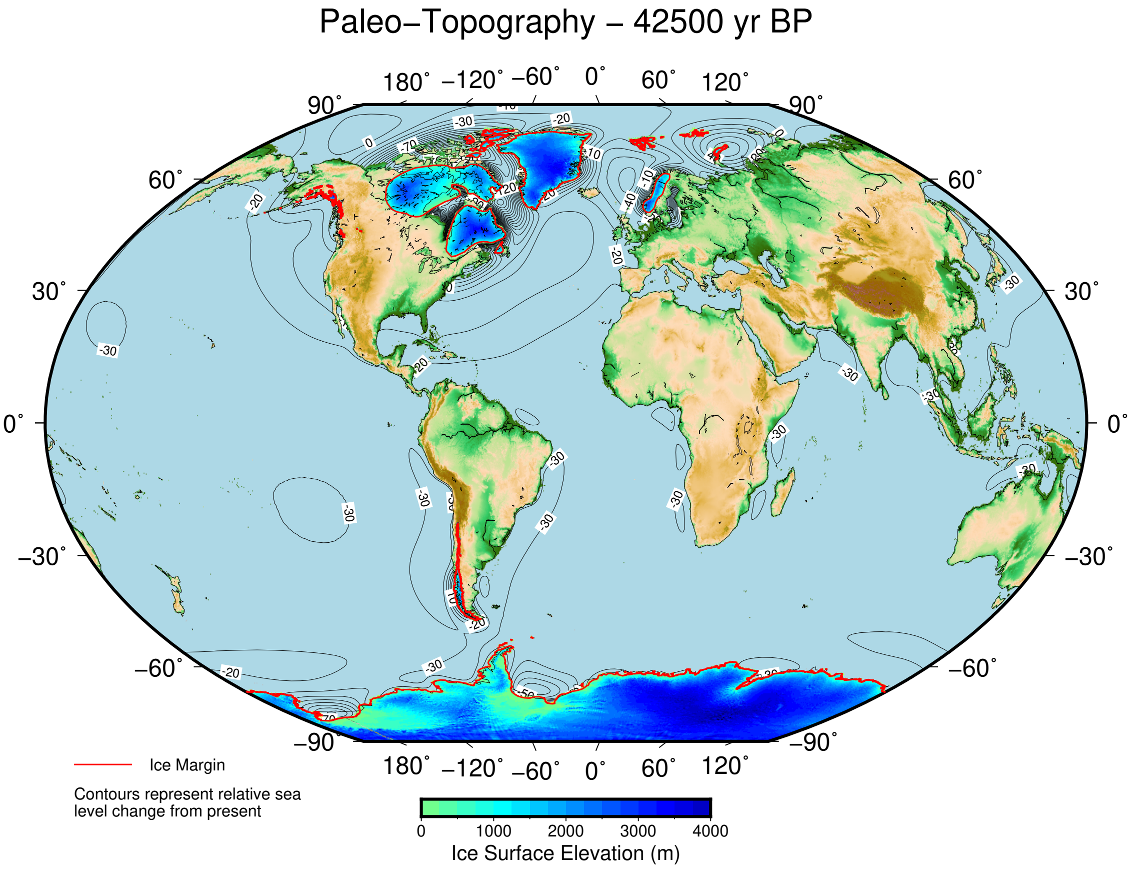

Supplementary Figure 10: Global paleo-topography at 42 500 yr BP, for the minimal MIS 3 ice sheet configuration. The contours represent sea level change relative to present.

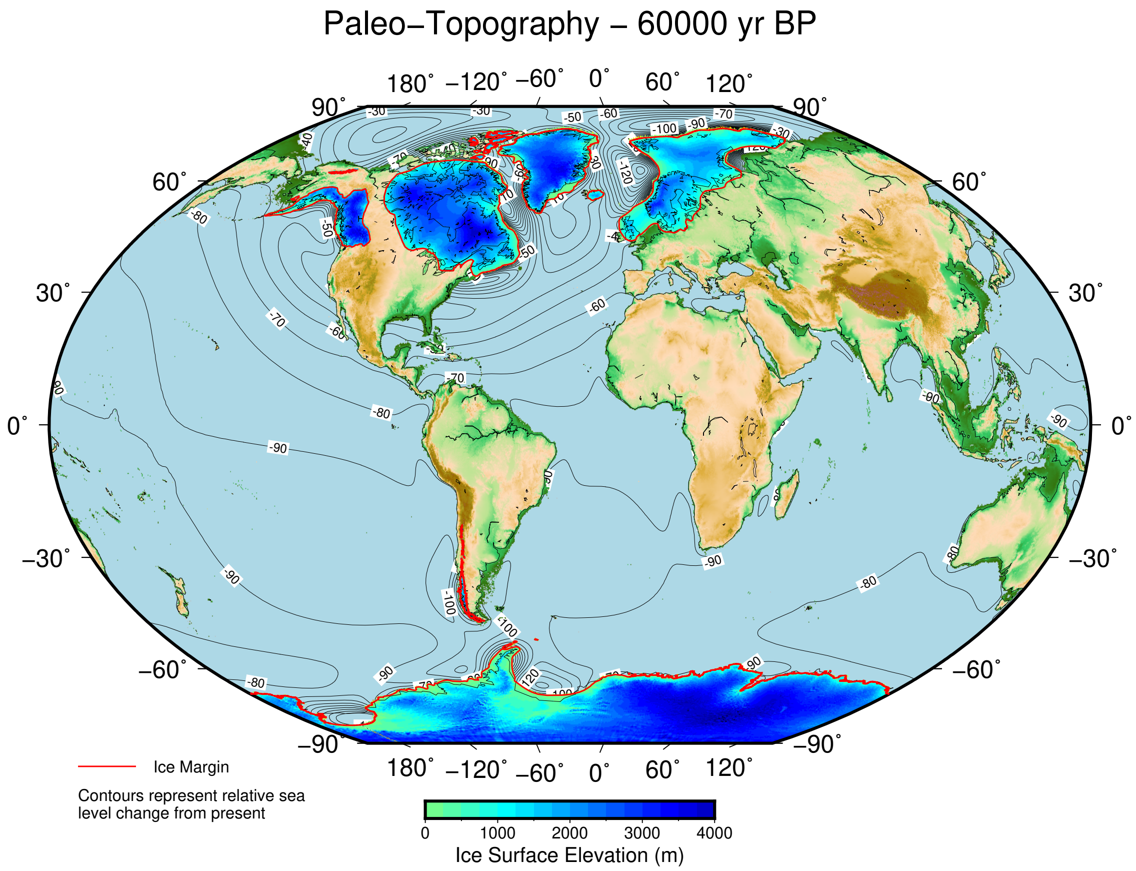

Supplementary Figure 11: Global paleo-topography at 60 000 yr BP, the maximum MIS 4 ice sheet configuration. The contours represent sea level change relative to present.

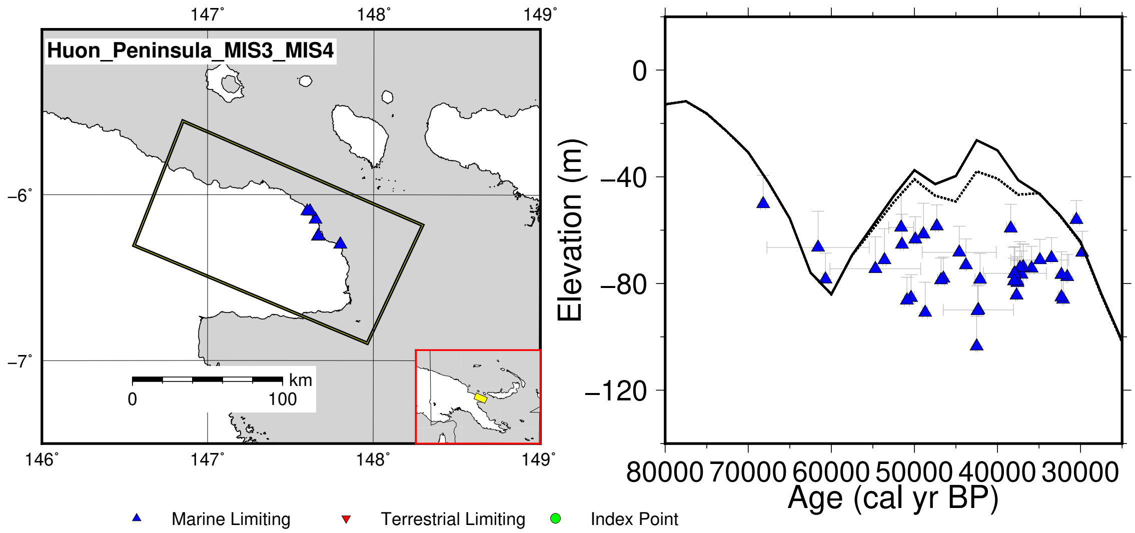

Supplementary Figure 12: Calculated sea level and MIS 3 and 4 sea level indicators in the Huon Peninsula, Papua New Guinea[5]. Solid lines are for the minimal MIS 3 scenario, while the dotted lines are for the maximal scenario.

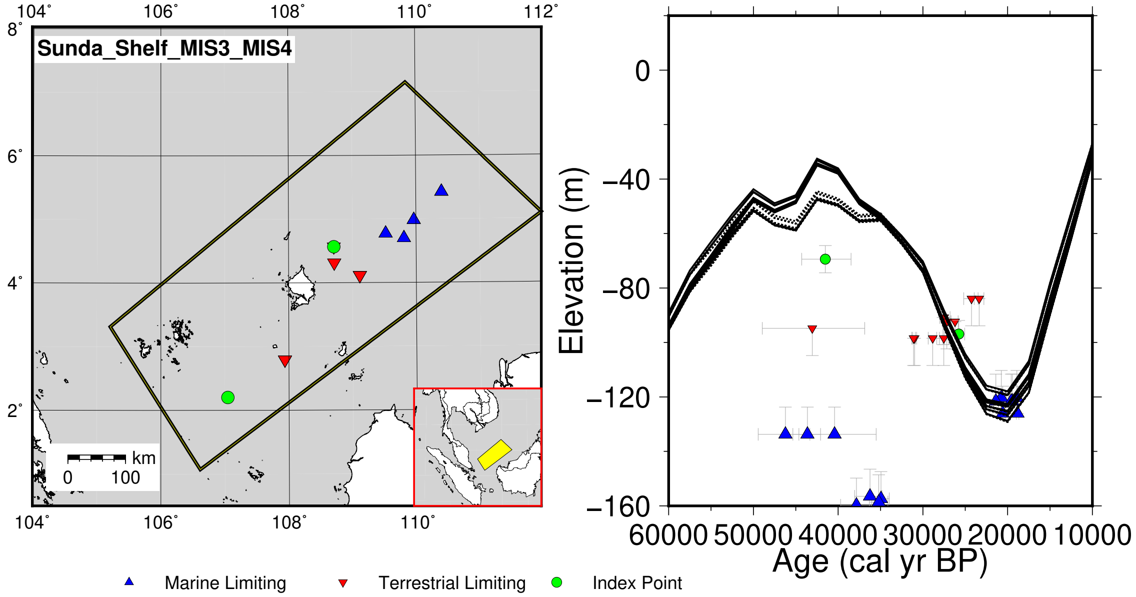

Supplementary Figure 13: Calculated sea level and MIS 3 sea level indicators in the Sunda Shelf, southeastern Asia[6,7]. Solid lines are for the minimal MIS 3 scenario, while the dotted lines are for the maximal scenario.

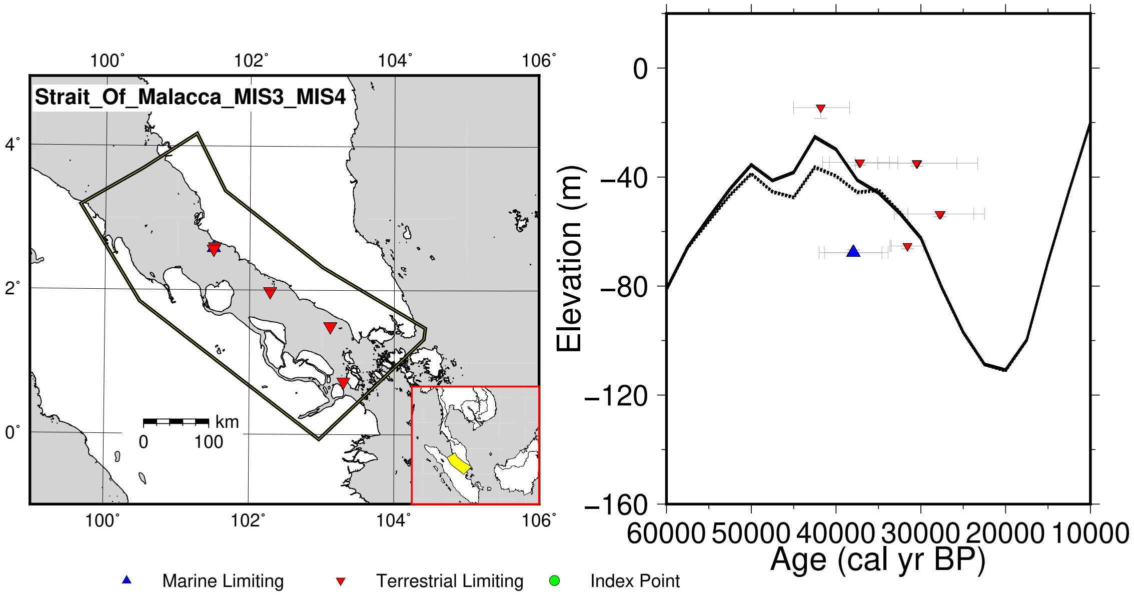

Supplementary Figure 14: Calculated sea level and MIS 3 sea level indicators in the Strait of Malacca, southeastern Asia[8]. Solid lines are for the minimal MIS 3 scenario, while the dotted lines are for the maximal scenario.

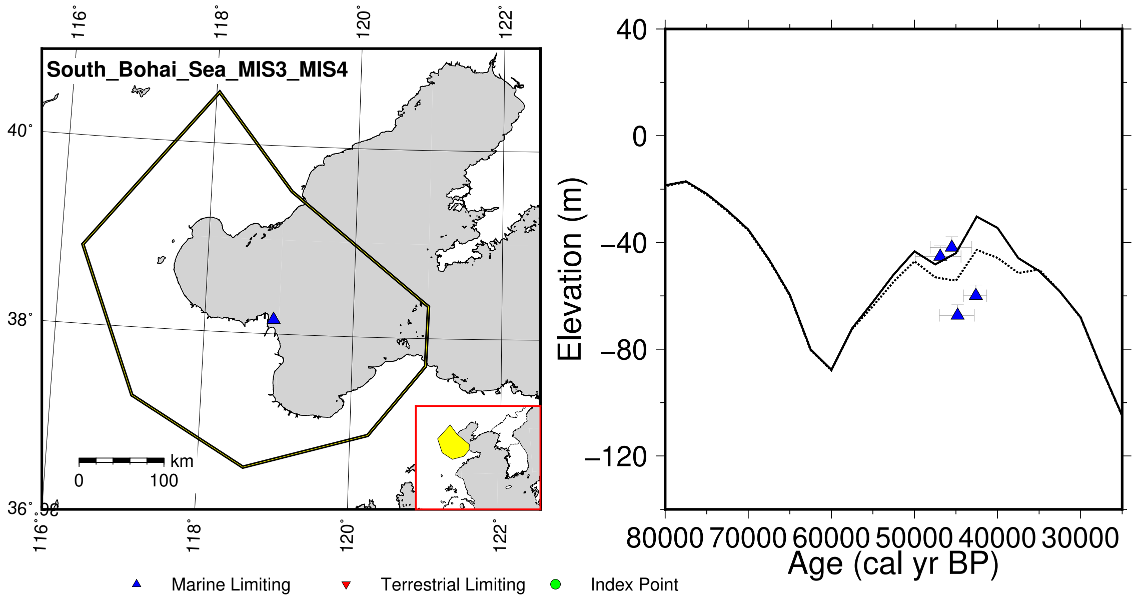

Supplementary Figure 15: Calculated sea level and MIS 3 sea level indicators in the South Bohai Sea, China[9]. Solid lines are for the minimal MIS 3 scenario, while the dotted lines are for the maximal scenario.

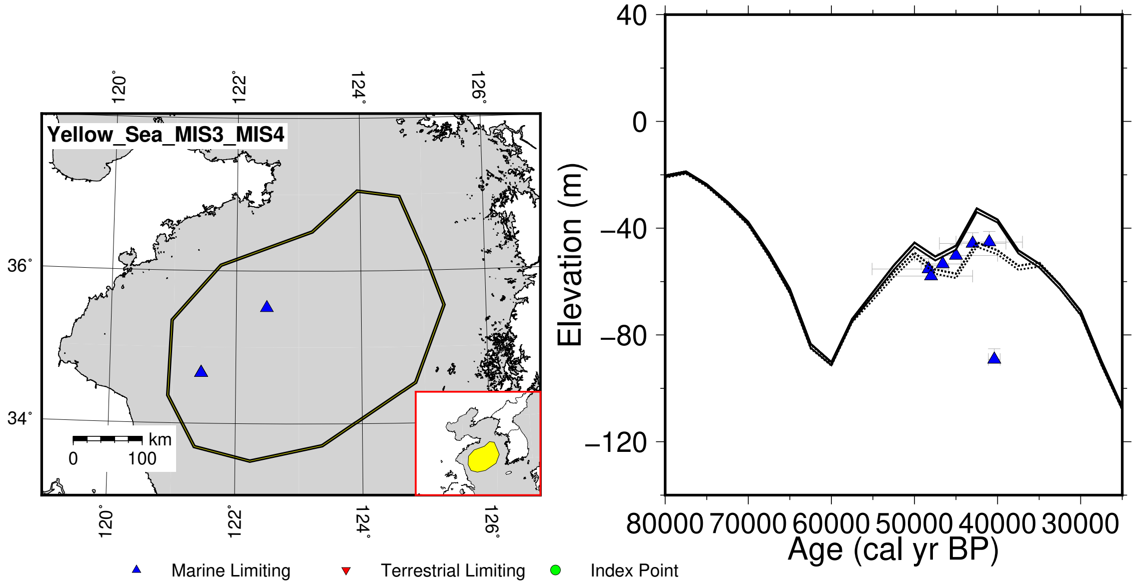

Supplementary Figure 16: Calculated sea level and MIS 3 sea level indicators in the Yellow Sea, eastern Asia[9]. Solid lines are for the minimal MIS 3 scenario, while the dotted lines are for the maximal scenario.

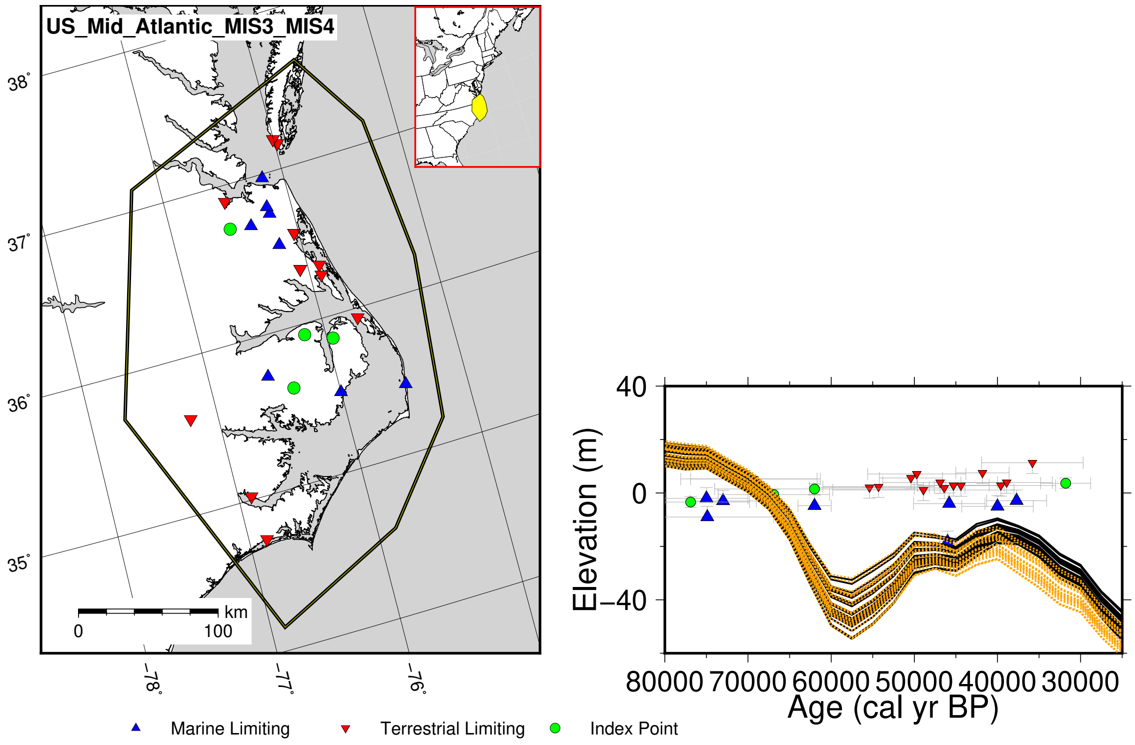

Supplementary Figure 17: Calculated sea level and MIS 3 and 4 sea level indicators in the mid-Atlantic coast of the United States[10]. Solid lines are for the minimal MIS 3 scenario, while the dotted orange lines are the maximal scenario.

Supplementary References

1. Vacchi, M. *et al.* Postglacial relative sea-level histories along the eastern Canadian coastline. *Quaternary Science Reviews* **201**, 124–146 (2018). doi:10.1016/j.quascirev.2018.09.043.
2. Wu, P. Sensitivity of relative sea levels and crustal velocities in Laurentide to radial and lateral viscosity variations in the mantle. *Geophysical Journal International* **165**, 401–413 (2006). doi:10.1111/j.1365-246X.2006.02960.x.
3. Batchelor, C. L. *et al.* The configuration of Northern Hemisphere ice sheets through the Quaternary. *Nature communications* **10**, 1–10 (2019). doi:10.1038/s41467-019-11601-2.
4. Licciardi, J., Clark, P., Jenson, J. & Macayeal, D. Deglaciation of a soft-bedded Laurentide Ice Sheet. *Quaternary Science Reviews* **17**, 427–448 (1998). doi:10.1016/S0277-3791(97)00044-9.
5. Hibbert, F. D. *et al.* Coral indicators of past sea-level change: A global repository of U-series dated benchmarks. *Quaternary Science Reviews* **145**, 1–56 (2016). doi:10.1016/j.quascirev.2016.04.019.
6. Hanebuth, T. J., Stattegger, K., Schimanski, A., Lüdmann, T. & Wong, H. K. Late Pleistocene forced-regressive deposits on the Sunda Shelf (Southeast Asia). *Marine Geology* **199**, 139–157 (2003). doi:10.1016/S0025-3227(03)00129-4.
7. Steinke, S., Kienast, M. & Hanebuth, T. On the significance of sea-level variations and shelf paleo-morphology in governing sedimentation in the southern South China Sea during the last deglaciation. *Marine Geology* **201**, 179–206 (2003). doi:10.1016/S0025-3227(03)00216-0.
8. Geyh, M., Streif, H. & Kudrass, H.-R. Sea-level changes during the late Pleistocene and Holocene in the Strait of Malacca. *Nature* **278**, 441 (1979). doi:10.1038/278441a0.
9. Pico, T., Mitrovica, J. X., Ferrier, K. L. & Braun, J. Global ice volume during MIS 3 inferred from a sea-level analysis of sedimentary core records in the Yellow River Delta. *Quaternary Science Reviews* **152**, 72–79 (2016). doi:10.1016/j.quascirev.2016.09.012.
10. Pico, T., Creveling, J. & Mitrovica, J. Sea-level records from the US mid-Atlantic constrain Laurentide Ice Sheet extent during Marine Isotope Stage 3. *Nature communications* **8**, 15612 (2017). doi:10.1038/ncomms15612.
